# Selection of an Endophytic *Streptomyces* sp. Strain DEF09 From Wheat Roots as a Biocontrol Agent Against *Fusarium graminearum*

**DOI:** 10.3389/fmicb.2019.02356

**Published:** 2019-10-11

**Authors:** Elena Maria Colombo, Andrea Kunova, Cristina Pizzatti, Marco Saracchi, Paolo Cortesi, Matias Pasquali

**Affiliations:** Department of Food, Environmental and Nutritional Sciences, University of Milan, Milan, Italy

**Keywords:** endophytes, cereal, BCA, toxigenic fungi, PGP, Fusarium head blight, Fusarium root rot, Fusarium foot rot

## Abstract

Selection of biological control agents (BCA) profits from an integrated study of the tripartite interactions occurring among the BCA, the plant and the pathogen. The environment plays a crucial role in the efficacy of BCA, therefore, the selection process shall utmost mimic naturally occurring conditions. To identify effective biocontrol strains against *Fusarium graminearum*, the major cause of Fusarium head blight (FHB) in wheat and deoxynivalenol (DON) accumulation in grains, a workflow consisting of *in vitro* and *in vivo* assays was set up. Twenty-one *Streptomyces* strains, 16 of which were endophytes of different plants, were analyzed. *In vitro* and *in vivo* tests characterized their plant growth promoting (PGP) traits. Biocontrol activity against *F. graminearum* was firstly assessed with a dual culture assay. An *in vivo* germination blotter assay measured Fusarium foot rot and root rot symptoms (FFR-FRR) reduction as well as growth parameters of the plant treated with the *Streptomyces* strains. A selected subset of *Streptomyces* spp. strains was then assessed in a growth chamber measuring FFR symptoms and growth parameters of the wheat plant. The approach led to the identification of an effective *Streptomyces* sp. strain, DEF09, able to inhibit FHB on wheat in controlled conditions by blocking the spread of the pathogen at the infection site. The results were further confirmed in field conditions on both bread and durum wheat, where DEF09 decreased disease severity up to 60%. This work confirms that FRR and FFR pathosystems can be used to identify BCA effective against FHB.

## Introduction

*Fusarium graminearum* is a major threat to wheat, leading to Fusarium foot rot (FFR) and Fusarium root rot (FRR) (Smiley and Patterson, 1996), as well as Fusarium head blight (FHB), the major cause of wheat losses (Goswami and Kistler, 2004). Losses are aggravated by the accumulation of deoxynivalenol (DON), an internationally regulated mycotoxin (Wegulo et al., 2015). The pathogenic behavior of the fungus has been widely studied at the spike level both from a molecular point of view (Ilgen et al., 2009; Lysøe et al., 2011) and from a physiopathological point of view (Boenisch and Schäfer, 2011). The pathogen, similarly to other known foot and root rot pathogens of wheat, such as *F. culmorum* (Scherm et al., 2013) and *F. pseudograminearum* (Chakraborty et al., 2006), has a specific pathway of infection and spread via roots (Wang Q. et al., 2015). Surprisingly, head blight, root, and foot rot caused by *F. graminearum* share most of the developmental steps of pathogenicity (Wang et al., 2018), including the DON synthesis (Covarelli et al., 2012).

*Streptomyces* spp. are well known Gram-positive bacterial symbionts of living organisms (Seipke et al., 2012), and can establish tight interactions with inner plant tissues (Coombs and Franco, 2003). They can act as plant growth promoters by producing phytohormones, facilitating nutrient uptake and inhibiting plant pathogens (Viaene et al., 2016; Vurukonda et al., 2018). They have been extensively investigated as a source of bioactive molecules (Watve et al., 2001) and *Streptomyces-*derived commercial products have been successfully applied for crop protection (Newitt et al., 2019). Indeed, several *Streptomyces* strains have been proposed as potential biocontrol agents against toxigenic fungi, including numerous *Fusarium* spp. causing diseases and mycotoxin accumulation in cereals (Nourozian et al., 2006; Palazzini et al., 2007; Yekkour et al., 2012; Jung et al., 2013).

Previous studies of *Streptomyces* strains effective against *F. graminearum* (Palazzini et al., 2007, 2017, 2018; Jung et al., 2013) did not assess their effect on the plant, despite the large arsenal of metabolites they produce may affect plant development. Moreover, often only *in vitro* tests are used to assess plant growth promoting (PGP) traits for strain characterization, and rarely BCA and PGP traits are evaluated in the presence of the host plant (Anwar et al., 2016).

One of the main limitations of historical biocontrol studies is that often the selection of strains is solely performed *in vitro*, which can result in the lack of activity in field conditions (Burr et al., 1996; Milus and Rothrock, 1997).

In an effort to set up a solid selection procedure of *Streptomyces* strains active against Fusarium head blight pathogens, the goal of this work was to characterize *Streptomyces* strains for both their PGP associated traits and their biocontrol activity, considering also tripartite interactions (plant, microorganism, pathogen) under different environmental conditions. To achieve this, the laboratory amenable pathosystems of FRR and FFR were used. This procedure proved successful in the identification of a *Streptomyces* sp. able to significantly limit FHB losses in field conditions.

## Materials and Methods

### *Streptomyces* Used in the Study

The collection of *Streptomyces* spp. maintained in the laboratory of Plant Pathology at the Department of Food, Environmental and Nutritional Sciences (DeFENS), University of Milan (Italy), hosts endophytic isolates from roots of different plants (Sardi et al., 1992) as well as from different sources (Table 1).

**TABLE 1:** *Streptomyces* strains used in the study.

				**Year of**	**Closest match as**		**GenBank**
**Strain**	**Source of**	**Environment of**	**Place of sample**	**sample**	**similarity % in**	**Completeness**	**accession**
**code**	**isolation**	**sample collection**	**collection**	**collection**	**EzBioCloud database**	**(%)**	**number**
DEF06	*Poa annua*	Golf course	Monticello (LC, Italy)	1989	99.50:*Streptomyces geysiriensis*	99.9	MK463961
DEF07^∗^	*Camellia japonica*	Greenhouse	Arona (NO, Italy)	1988	99.36:*Streptomyces venetus*	100	MK412001
DEF08	*Polyporus* sp.	Plane tree	Monza (Italy)	1980	100:*Streptomyces coelicoflavus*	99.9	MK463962
DEF09^∗^	*Triticum aestivum*	Botanic garden	Milano (Italy)	1989	99.93%: *Streptomyces fulvissimus*	100	MK412002
DEF13^∗^	*Polyporus* sp.	Plane tree	Monza (Italy)	1980	100:*Streptomyces coelicoflavus*	100	MK412004
DEF14^∗^	*Arundo* sp.	Lake shores	Ansedonia (GR, Italy)	1996	99.93%: *Streptomyces fulvissimus*	100	MK412005
DEF15^∗^	*Secale cereale*	Botanic garden	Milano (Italy)	1989	100:*Streptomyces setonii*	100	MK412006
DEF16^∗^	*Zea mays*	Cultivated field	Cantù (CO, Italy)	1985	99.71:*Streptomyces albidoflavus*	100	MK412007
DEF17	*Hordeum vulgare*	Botanic garden	Milano (Italy)	1989	99.50:*Streptomyces tanashiensis*	100	MK463963
DEF18	*Triticum aestivum*	Botanic garden	Milano (Italy)	1989	100:*Streptomyces setonii*	100	MK463964
DEF19^∗^	*Camellia japonica*	Greenhouse	Arona (NO, Italy)	1988	99.37:*Streptomyces venetus*	100	MK412008
DEF20^∗^	*Carex* sp.	Lake shores	Mergozzo (NO, Italy)	1989	99.37:*Streptomyces venetus*	100	MK412009
DEF21	*Zea mays*	Cultivated field	Cantù (CO, Italy)	1985	100:*Streptomyces setonii*	100	MK463965
DEF31	*Homo sapiens*	Crypt	S. Fruttuoso (GE, Italy)	1960	100:*Streptomyces calvus*	100	MK463966
DEF33	unknown plant	Natural environment (savanna)	Canaima (Venezuela)	1993	99.57:*Streptomyces corchorusii*	100	MK463967
DEF39^∗^	*Secale cereale*	Botanic garden	Milano (Italy)	1989	100:*Streptomyces setonii*	100	MK412014
DEF40	*Secale cereale*	Botanic garden	Milano (Italy)	1989	100:*Streptomyces costaricanus*	100	MK463968
DEF41^∗^	unknown plant	Natural environment (savanna)	Canaima (Venezuela)	1993	100:*Streptomyces costaricanus*	100	MK412015
DEF46	*Homo sapiens*	Crypt	S. Fruttuoso (GE, Italy)	1960	100:*Streptomyces calvus*	100	MK463969
DEF47^∗^	unknown plant	Natural environment (savanna)	Canaima (Venezuela)	1993	100:*Streptomyces costaricanus*	100	MK412018
DEF48^∗^	*Zea mays*	Cultivated field	Cantù (CO, Italy)	1985	99.36:S*treptomyces venetus*	100	MK412019

*^∗^Strains identified in Colombo et al. (2019). Underlined strains were not originally isolated as endophytes.*

The twelve most active strains able to significantly inhibit FFR or FRR caused by *F. graminearum* (activity above 40%), identified in a comparative work of *in vitro* screening methods (Colombo et al., 2019), were selected for this study together with new isolates of diverse origin identified in this work (Table 1). Overall, twenty-one strains were used.

### *Streptomyces* Identification

Bacterial isolates DEF07, DEF09, DEF13, DEF14, DEF15, DEF16, DEF19, DEF20, DEF39, DEF41, DEF47, and DEF48 were identified in Colombo et al. (2019).

DNA from isolates DEF06, DEF08, DEF17, DEF18, DEF21, DEF31, DEF33, DEF40, and DEF46 was extracted following the method described by Sun et al. (2014). Briefly, a single bacterial colony was transferred to a sterile 1.5 mL tube containing 27 μL Tris (10 mM)-EDTA (1 mM) (pH 7.6); then, 3 μL KOH (0.4 M) – EDTA (10 mM) were added and incubated at 70°C for 5 min. Next, 3 μL Tris-HCl (10 mM) (pH 4.0) were added to adjust the pH of the lysate. The lysate was used directly as a DNA template for the PCR amplification. 16S rRNA primers (Turner et al., 1999) used were 27F (5′-AGAGTTTGATCCTGGCTCAG-3′) and rP2 (5′-ACGGCTACCTTGTTACGACTT-3′). PCR was performed in a total volume of 50 μL, which contained 0.3 μL of GoTaq^®^ DNA Polymerase 5 U/μL (Promega, United States), 10 μL of Green GoTaq^®^ Reaction Buffer 5X (Promega, United States), 1 μL of 10 mM dNTP (Promega, United States), 1 μL of 10 μM primer forward, 1 μL of 10 μM primer reverse, 1 μL of template DNA and nuclease free water. The reaction conditions were initial denaturation at 95°C for 5 min, followed by 35 cycles of denaturation at 95°C for 20 s, annealing at 56°C for 30 s and extension at 72°C for 90 s. A final extension was performed at 72°C for 7 min. Reaction products were separated by electrophoresis on a 1.5% agarose gel containing ethidium bromide and visualized under UV light. The PCR products were sequenced in both directions (Eurofins Genomics, Germany) using 27F and rP2 primers and two internal primers 16s_p692f (5′-AATTCCTGGTGTAGCGGT-3′) and 16s_p782r (5′-ACCAGGGTATCTAATCCTGT-3′). Assembled sequences were obtained with Geneious Prime 2019 (Biomatters, United States). EzBioCloud database was used to identify the strains based on 16S rRNA sequences (Yoon et al., 2017).

### Preparation of Bacterial Inoculum

Spores were collected after 2 weeks of incubation at 24°C on Czapek Yeast Extract medium (CZY: 35 g/L czapek dox broth, Difco Laboratories, United States; 2 g/L yeast extract, Difco Laboratories, United States; 15 g/L agar, Amresco, United States; pH 6.5) scraping the surface of the colonies with a sterile loop and 5 mL of 10% sterile glycerol (ICN Biomedicals, United States) + 0.01% Tween20 solution (Sigma-Aldrich, United States). The concentration was determined using a hemocytometer and adjusted to 10^7^ spores/mL. Small aliquots were then stored at −20°C.

### Antibiosis Assay

The antibiosis assay was performed using one medium and 22 treatments (21 *Streptomyces* strains + one water control). Three replicates were prepared. Briefly, 10 μL of *Streptomyces* spp. agar-spore suspension (10^6^ spores/mL) or sterile water were inoculated on a Petri plate containing Wheat Meal Agar (WMA; Colombo et al., 2019). After 3 days, a plug of agar-mycelium (6 mm diameter) was taken from the edge of an actively growing colony of *F. graminearum* Fg8/1 (Boenisch and Schäfer, 2011) and inoculated upside down in the center of the plate at 25 mm distance from the bacterial strain. After a period of incubation (3 days at 24°C in the dark), the antagonistic activity was assessed measuring the mycelial radial growth of the pathogen in the control (R1) and in the presence of the antagonist (R2). The percentage of mycelium growth inhibition compared to the control was calculated according to the Equation (1):

(1) (R1-R2)/R1×100

### Screening for PGP Traits *in vitro*

The twenty-one strains involved in the study were screened for PGP characteristics. Indole acetic acid (IAA) production, tricalcium phosphate solubilization, siderophore and chitinase production, starch hydrolysis, nitrate reduction and growth in presence of salt were determined following the procedures described below.

The IAA production was evaluated following the method described in Bano and Musarrat (2003). Ten μL of *Streptomyces* strain spore suspension (1 × 10^6^ spores/mL) were inoculated in 5 mL of CZY broth (35 g/L czapek dox broth, Sigma-Aldrich, United States; 2 g/L yeast extract, Difco Laboratories, United States; pH 6.5) adding 500 μg/mL of tryptophan (Sigma-Aldrich, United States). Three replicates were prepared. After a period of incubation (24°C for 10 days at 125 rpm), the liquid cultures were centrifuged (10,000 rpm, 10 min, 15°C) and 2 mL of supernatant were collected and mixed with 100 μL of 10 mM orthophosphoric acid (Carlo Erba Reagents, Italy) and 4 mL of Salkowski reagent (1 mL 0.5 M FeCl_3_, Sigma-Aldrich, United States; 49 mL 35% HClO_4_, Sigma-Aldrich, United States). The samples were incubated at room temperature for 20 min in the dark. The development of pink color indicated the IAA production. The absorbance of the samples was measured with a spectrophotometer (Perkin-Elmer Lambda 20, United States) at 530 nm. The concentration of IAA produced was calculated based on a standard curve of IAA obtained in the range of 1–50 μg/mL.

The ability to solubilize tricalcium phosphate was assessed in Petri plates (90 mm diameter) containing National Botanical Research Institute’s Phosphate growth medium (10 g/L glucose, Sigma-Aldrich, United States; 5 g/L Ca_3_(PO_4_)_2_, Sigma-Aldrich, United States; 5 g/L MgCl_2_ × 6H_2_O, Carlo Erba Reagents, Italy; 0.25 g/L MgSO_4_ × 7H_2_O, Carlo Erba Reagents, Italy; 0.2 g/L KCl, Merck, Germany; 0.1 g/L (NH_4_)_2_SO_4_, Carlo Erba Reagents, Italy; 15 g agar, Amresco, United States; pH 7) as described in Nautiyal (1999), inoculated with 10 μL of spore suspension (1 × 10^6^ spores/mL). Three replicates were prepared. After an incubation period (24°C for 14 days), the halo was visually assessed. The halo width of 1 mm was marked with +, lack of halo was marked with −.

Siderophore production was observed using Chrome Azurol S agar overlay method (O-CAS) as described by Pérez-Miranda et al. (2007). The strains were grown on a modified CZY medium without iron (pH 6.5) for siderophore production. Ten μL of agar-spore suspension (10 μL of spore suspension in 90 μL of 0.2% water agar) were inoculated in the center of a Petri plate (90 mm diameter) and kept for 14 days at 24°C. Subsequently, 15 mL of Chrome Azurol S agar were cast upon culture agar plates (CAS agar: 60.5 mg/L Chrome Azurol S, Sigma-Aldrich, United States; 72.9 mg/L hexadecyltrimethyl ammonium bromide, Honeywell Fluka, Buchs, Switzerland; 30.24 g/L piperazine-1,4-bis(2-ethanesulfonic acid), Sigma-Aldrich, United States; 10 mL 1 mM FeCl_3_ × 6H_2_O in 10 mM HCl, Sigma-Aldrich, United States; 9 g/L agar, Amresco, United States). Three replicates were prepared. In addition, a negative control was prepared using normal CZY medium. After 1 day of incubation at room temperature in the dark, the change of color around the colony (from blue to orange) indicated the siderophore production. The orange halo (D) and colony (d) diameters were measured and the production of siderophores was calculated according to the Equation (2):

(2) (D-d)/2

Chitinase production (Kuddus and Ahmad, 2013) was assessed using chitin medium (6 g/L Na_2_HPO_4_, Carlo Erba Reagents, Italy; 3 g/L KH_2_PO_4_, Carlo Erba Reagents, Italy; 1 g/L NH_4_Cl, Carlo Erba Reagents, Italy; 0.5 g/L NaCl, Carlo Erba Reagents, Italy; 0.05 g/L yeast extract, Difco Laboratories, United States; 1% (w/v) colloidal chitin; 15 g/L agar, Amresco, United States; pH 6.5). Colloidal chitin was prepared adding 20 g of chitin (Sigma-Aldrich, United States) to 300 mL of 37% HCl (Merck, Germany). The chitin-HCl solution was kept for 60 min at 30°C in continuous stirring and then precipitated adding 1 L of cold water. In order to allow the precipitation of the colloidal particles, the material was kept at 4°C overnight and then collected by filtration on filter paper, washing with deionized water to bring up the pH at 6. Petri dishes (45 mm diameter) containing chitin medium were inoculated with 10 μL of agar-*Streptomyces* spore suspension (1 × 10^6^ spores/mL). Three replicates were prepared. After an incubation period (24°C for 14 days), the halo (D) and the colony (d) diameters were measured and the capacity to degrade chitin was expressed using Equation (2).

The ability to hydrolyze starch (Shirling and Gottlieb, 1966) was evaluated streaking a single colony of each *Streptomyces* strain on Petri dishes (90 mm diameter) containing ISP Medium 4 (Difco Laboratories, United States, pH 7.2) added with 10 g/L of soluble starch (Difco Laboratories, United States) and 1 mL of trace salts solution (per 100 mL: 100 mg FeSO_4_ × 7H_2_O Merck, Germany; 100 mg MnCl_2_ × 4H_2_O Carlo Erba Reagents, Italy; 100 mg ZnSO_4_ × 7H_2_O Carlo Erba Reagents, Italy). Three replicates were prepared. After a period of incubation (24°C for 14 days), the presence of the hydrolysis halo around the colonies determined the amylase activity.

The nitrogen reduction capability (Shirling and Gottlieb, 1966) was assessed by inoculating glass tubes containing 5 mL Bacto-Nitrate medium (13 g/L nutrient broth, Oxoid, Italy; 2 g/L KNO_3_, Carlo Erba Reagents, Italy; 2 g/L bacto agar, Difco Laboratories, United States; pH 6.5) with a single colony of each strain. Three replicates were prepared. After an incubation period (24°C for 14 days), 200 μL of nitrate reagent A (α-naphthylamine, Sigma-Aldrich, United States) and B (sulfanilic acid, Sigma-Aldrich, United States) were added in each tube. The presence of nitrite was confirmed by the development of a red color after the formation of a diazonium salt caused by the reaction between the reagents A and B.

High salt concentration growth was evaluated streaking single colonies on Bennet’s agar medium (Jones, 1949) (1 g/L yeast extract, Difco Laboratories, United States; 0.8 g/L lab-lemco, Oxoid, Italy; 10 g/L glucose, Sigma-Aldrich, United States; 2 g/L casitone, Difco Laboratories, United States; 15 g/L agar, Amresco, United States; pH 6.5) added with 3.5% or 7% (w/v) of NaCl (Carlo Erba Reagents, Italy). Three replicates were prepared. After 14 days of incubation at 24°C, the growth of the strains in Petri dishes (45 mm diameter) was evaluated in comparison with control plates (0% NaCl).

### Seed Treatments and Blotter Assay Germination

The twenty-one strains were tested for their potential growth promoting and biocontrol activities against FRR and FFR. Seeds of *Triticum aestivum* L. cv. Bandera were surface-sterilized in 0.7% sodium hypochlorite for 5 min and then rinsed 3 times in sterile water. In sterile Petri dishes, seeds (*N* = 40) were inoculated with 1 mL of *Streptomyces* strain spore suspension (10^7^ spores/mL) and dried under the laminar flow hood. Control seeds were treated with 1 mL of deionized sterile water. For biocontrol experiments, seeds (*N* = 40) were treated in the same way. After 4 days, the seedlings were inoculated with an agar-mycelium plug (6 mm diameter) taken from the edge of an actively growing *F. graminearum* Fg8/1 colony and inoculated upside down on the roots at a 10 mm distance from the seed. The assay took place in sterile glass dishes as seed trays (diameter 150 mm). In each dish, a filter paper was placed and soaked with 10 mL deionized sterile water before sowing. For each condition, four glass dishes containing 10 seeds arranged in three rows were prepared. The germination of the seeds followed the conditions described in Covarelli et al. (2013). Briefly, the dishes were placed at 5°C in the dark for 24 h simulating a period of vernalization and then moved at 20°C in the dark. Three days after seed bacterization, dishes were placed in a growth chamber (21°C, 16 h photoperiod using fluora lamp osram L36W/77). Seedlings were watered with sterile deionized water every 2 days.

### Evaluation of PGP and Biocontrol Effects in Germination Blotter Assay

Germination was assessed after 2 days of incubation in the growth chamber, when seeds were still in the dark to simulate normal germination process, while root and seedling length as well as root number were assessed after 3 and 10 days. At the 10th day, seedlings were dried and the root and shoot dry weight was assessed.

The biocontrol potential of the *Streptomyces* spp. against Fusarium root rot (FRR) and foot rot (FFR) was evaluated using *F. graminearum* Fg8/1 infected seedlings. Four days after pathogen inoculation the FRR was measured on 20 roots as necrosis development. FRR data were reported as millimeters of necrosis extension. Percentages of necrosis inhibition were calculated using measurements of necrosis on the control (CN) and on the treated seedlings (TN) using the Equation (3):

(3) (CN-TN)/CN×100

Six days after seed bacterization, FFR was evaluated by scoring the symptoms at the crown level on 20 seedlings (Covarelli et al., 2013) with a 0–4 scale (0 = symptomless; 1 = slightly necrotic; 2 = moderately necrotic; 3 = severely necrotic; 4 = completely necrotic) (Colombo et al., 2019). The FFR disease severity was calculated for each treatment using the Equation (4):

(4) [∑(disease grade×number of plants in each grade)/(total number of plants×the highest disease grade)]×100

The ability of the antagonists to reduce symptom development was assessed with the Equation (5):

(5) (DC-DT)/DC×100

DC and DT were the disease severity in the control and the treated seedlings, respectively.

In addition, shoots from infected and control seedlings were dried and their weight was measured.

### *Streptomyces* Biocontrol Activity Against FFR in Soil Substrate

Seed bacterization with *Streptomyces* spp. spore suspension was carried out as described above with strains that showed promising BCA features *in vitro* (DEF07, DEF09, DEF19, DEF20, DEF39, DEF47, and DEF48). DEF08 was used as a negative control, as it showed no FFR inhibition in the previous test. Conidia of *F. graminearum* Fg8/1 were produced in CMC medium (15 g/L carboxymethyl-cellulose, Sigma-Aldrich, United States; 1 g/L NH_4_NO_3_, Carlo Erba Reagents, Italy; 1 g/L KH_2_PO_4_, Carlo Erba Reagents, Italy; 0.5 g/L MgSO_4_ × 7H_2_O, Carlo Erba Reagents, Italy; 1 g/L yeast extract, Difco Laboratories, United States; pH 6.5). Conidia were collected as described in Breakspear et al. (2011) after 5 days of incubation by filtering cultures through one layer of Miracloth (Calbiochem, United States) and centrifuging the filtrate for 10 min at 3000 rpm. Supernatant was discarded and the pelleted conidia were washed twice with sterile water (centrifuge 10 min, 3000 rpm).

Twenty inoculated seeds for each treatment (water or *Streptomyces* spp. spore suspension) were placed in sterile glass dishes (diameter 150 mm) to allow their germination at room temperature. After 6 days, wheat seedlings were transplanted in polystyrene seed trays (32 × 52 × 5.5 cm) containing sterile substrate (1:1 ratio of Irish peat and sand, pH 6.5, EC 0.2 dS/m, density 340 kg/m^3^, porosity 89% v/v, Vigorplant, Italy) watered with tap water. After that, roots were inoculated with one agar-mycelium plug (6 mm diameter) taken from a colony of *F. graminearum* Fg8/1 grown on V8 medium (Spanu et al., 2012). In addition, 1 mL of *F. graminearum* Fg8/1 conidia (1 × 10^6^ conidia/mL) or a mixture of *F. graminearum* Fg8/1 (1 × 10^6^ conidia/mL) + *Streptomyces* (5 × 10^6^ spores/mL) was added to control plants or already bacterized plants, respectively (Simpson et al., 2000). Seedlings inoculated only with *Streptomyces* strains, as well as a non-inoculated seeds (water-only) to be used as controls were prepared.

Plants were grown in a growth chamber (Conviron, Winnipeg, Canada) at 24°C, 55% relative humidity and 15 h photoperiod, watered with tap water every 2 days. After 20 days, FFR disease symptoms were visually evaluated using a 0–4 scale (0 = symptomless; 1 = slightly necrotic; 2 = moderately necrotic; 3 = severely necrotic; 4 = completely necrotic) (Supplementary File 1). The FFR disease severity and protection level were calculated for each treatment using the Equations (4) and (5), respectively. Dried shoot weight of the infected seedlings was also assessed.

### *Streptomyces* spp. Re-isolation From Inner Root Tissues and Evaluation of PGP Effect in Soil Substrate Assay

Control plants and plants inoculated only with *Streptomyces* (no-*Fusarium* inoculation) from the previous test were harvested 20 days after transplant and washed in sterile water to remove the excess soil. Shoot length and dried weight of wheat plants were assessed for each treatment.

For inner root tissue analysis, 10 seedlings for each treatment were selected and cut at the base. The roots were washed and surface sterilized with propylene oxide (Sigma-Aldrich, United States) for 1 h (Sardi et al., 1992). Subsequently, 10 or 15 root pieces were cut in sterile conditions and placed in water agar medium (WA) containing 15 g/L agar (Amresco, Italia), 25 mg/L nalidixic acid (Sigma-Aldrich, United States), 50 mg/L nystatin (Sigma-Aldrich, United States), and 50 mg/L cycloheximide (Sigma-Aldrich, United States). Plates were incubated for 7 days at 24°C. Growth of *Streptomyces* spp. colonies on the plate was visually observed using a microscope. Morphological examination was carried out to confirm the re-isolation. Roots not inoculated with *Streptomyces* strains were used as negative control and subjected to the same procedure to check the presence of *Streptomyces* spp.

### Evaluation of Biocontrol Activity Against FHB in Growth Chamber

Spring wheat (*Triticum aestivum* L.) cv. Bandera was cultivated in growth chamber following the procedure described in Watson et al. (2018) to speed up the plant development to reach anthesis in approximately 2 months. Briefly, seeds were sterilized as described before and placed in sterile glass dishes (diameter 150 mm) to allow their germination. After 3 days at 4°C they were placed at room temperature for another period before sowing them in pots (21 × 13 × 15.5 cm, five seeds per pot) containing non-sterilized Irish and Baltic peat-based growth substrate (pH 6, EC 0.25 dS/m, density 120 kg/m^3^, porosity 90% v/v, Vigorplant, Italy). The lighting was set to 12 h light/12 h dark cycle for 4 weeks and then increased to an 18 h light/6 h dark photoperiod using fluora lamp osram L36W/77 until complete spike development. The temperature of the growth chamber was set at 18°C. *Fusarium graminearum* strain PH1 (Seong et al., 2009) was used to inoculate wheat heads. Bacterial spores of DEF09, which showed consistent biocontrol efficacy under all tested conditions, were prepared in CZY as described previously and *F. graminearum* conidia were prepared in CMC medium. The day of the treatment spores and conidia were collected and mixed with 0.01% Tween 20 (Sigma-Aldrich, United States) immediately before head inoculation. The final concentration of the mixture was 1 × 10^7^ spore/mL for DEF09 and 1 × 10^6^ conidia/mL for PH1. Ten μL of this mixture was used to inoculate the fifth centrally located spikelet from the bottom at anthesis. Three replicates were prepared for each treatment and arranged in a randomized block design. Three head treatments were performed: (1) *F. graminearum*, (2) *F. graminearum* + *Streptomyces* sp. DEF09, (3) Control (sterile distilled water + 0.01% Tween 20). Each spike was sealed in a plastic bag for 3 days. The FHB severity was visually estimated using a 0–100% scale 7 days after the treatment (Stack and McMullen, 1998). The average of FHB infection level was scored and the protection level calculated using the Equation (5).

### Evaluation of Biocontrol Activity Against FHB in Field Conditions

In order to further verify the biocontrol effect of DEF09 against FHB under complex environmental conditions, a field trial was performed. Field trial was conducted in Travacò Siccomario, Pavia (45°08′50.1^″^N 9°09′20.0^″^E, Italy), during the growth season 2019. The spring wheat (*Triticum aestivum* L.) cv. Bandera and the durum wheat (*Triticum turgidum* L. ssp. *durum*) cv. Claudio (both susceptible to *F. graminearum*) were sown with a 200 kg/ha density at the end of October 2018 on a loamy soil (Sand 31.2%, Silt 47.5%, Clay 21.3, cation exchange capacity 21.3 cmol^+^ kg DM^–1^, total organic carbon 1.51% DM, soil organic matter 2.60% DM, total Kjeldal nitrogen 0.19% DM, C/N ratio 7.95, P_2_O_5_ Olsen 87 mg kg DM^–1^, where DM stands for dry matter) with neutral pH (7.1). The field was previously cultivated with soybeans. Nitrogen fertilization was 30 kg/ha at the sowing and 50 kg/ha before booting. Weeding was carried out with Arianne II (Corteva, Italy) the 15/03/2019 at a dose of 3.5 l/ha. DEF09 spores and *F. graminearum* PH1 conidia were freshly produced in the laboratory as described above and collected at the day of field inoculation. Biocontrol assays started at wheat anthesis stage (beginning of May). Flowering period of the two cultivars differed by 7 days. Spores were kept on ice (max 2 h) until inoculation. Thirty plants at anthesis stage were selected for each treatment. Controls included: (a) conidia of *F. graminearum* PH1 2 × 10^6^ conidia/mL, (b) spores of *Streptomyces* DEF09 2 × 10^7^ spores/mL; (c) sterile distilled water + 0.01% Tween 20 (Sigma-Aldrich, United States). The treatment consisted of bacterial suspension and conidia + 0.01% of Tween 20 (Sigma-Aldrich, United States), mixed before head inoculation. The final concentration of the mixture was 2 × 10^7^ spores/mL for DEF09 and 2 × 10^6^ conidia/mL for PH1. Ten μL of spore suspension per treatment were used to inoculate a single, centrally located spikelet at anthesis. Inoculation was arranged in a randomized block design. Wheat heads were evaluated after 30 days. The infected spikelets were counted and FHB disease severity was visually estimated using a 0–100% scale (Stack and McMullen, 1998) for both wheat cultivars. Protection level of DEF09 treatment was assessed using the Equation (5).

### Statistical Analysis

Statistical analyses were performed using R software, version 3.5.1 (R Core Team, 2018), unless stated otherwise. To understand the effect of *Streptomyces* treatments on plant development and on FRR a Kruskal–Wallis test was applied, followed by a Dunn’s test with Bonferroni’s correction of the *P-*values to control the experiment-wise error rate (R package “dunn.test,” Dinno, 2017). Unless stated otherwise *P* < 0.05 was considered significant.

In order to identify treatments able to protect seedlings from FFR symptom development in comparison to the untreated control, a Fisher’s test was performed pooling the FFR symptoms in two groups [asymptomatic (class 0) or symptomatic (classes 1–4)]. Moreover, to assess also differences within the range of symptomatic seedlings, an additional Fisher’s test was carried out comparing the group of mild symptomatic (classes 1–2) with the severely diseased group (classes 3–4). *P* < 0.01 for both tests was considered significant.

CORREL function [CORREL(x, y)] in Microsoft Excel was used to determine the correlation coefficient between the results of the dual culture assay and chitinase activity, FRR protection and FFR protection. The Equation (6) for correlation coefficient is:

(6) $$\sum \left(x-\bar{x}\right)\,\left(y-\bar{y}\right)/\surd \,\sum {\left(x-\bar{x}\right)}^{2}\,\sum {\left(y-\bar{y}\right)}^{2}$$

For field trials, a two-group analysis (Mann–Whitney test) using Estimation stats (Ho et al., 2018) was conducted for each cultivar on the number of diseased spikelets of control (PH1) and treatment (PH1 + DEF09). The results are presented on a Gardner-Altman estimation plots.

## Results

### Screening for *Streptomyces* Biocontrol and PGP Activities *in vitro*

Identification of the nine isolates not identified in a previous study (Colombo et al., 2019) by 16S rRNA confirmed that all 21 strains belong to *Streptomyces* spp. (Table 1).

Results of *in vitro* tests for physiological and biochemical features directly or indirectly involved in plant growth promotion are reported in Table 2. Chitinase activity is widespread among all strains, but it is not correlated with the ability to reduce *F. graminearum* mycelium development (*r* = 0.22). Low amount of IAA production was recorded at the tested conditions, except for DEF09 and DEF33 that produced 2.50 ± 0.04 and 7.51 ± 0.00 μg/mL of IAA, respectively. Siderophore production was observed for DEF06, DEF17, DEF18, and DEF46. The radius of the halo ranged from 3 to 36 mm and DEF46 showed the widest halo of siderophore production on CAS agar. Only DEF06, DEF17, and DEF21 were able to solubilize tricalcium phosphate on NBRIP medium. Starch hydrolysis was common among the strains except for DEF09, DEF13, DEF20, and DEF41. Eleven strains reduced nitrate at the tested conditions (Table 2). All strains except DEF33 were able to grow at 3.5% salt in the medium and 71% grew even at 7% salt concentration. The antifungal activity of the *Streptomyces* strains against *F. graminearum* Fg8/1 in dual culture assay varied from 41% inhibition for DEF31 to 70% inhibition for DEF07, DEF19, DEF20, and DEF48 (Table 3).

**TABLE 2:** The screening of plant growth promotion traits *in vitro* and *in planta* (germination blotter or soil substrate assays).

	***In vitro* assays**								**Germination blotter assay**					**Soil substrate assay**	
**Treatment**	**IAA production (μg/mL) ± SD**	**Phosphate solubilization activity**	**Siderophore production (mm) ± SD**	**Chitinase activity (mm) ± SD**	**Starch hydrolysis**	**Nitrate reduction**	**Growth at high salt concentration**		**Germinated seeds (%) per blotter ± SD**	**Root length (mm) per plant ± SD**	**Shoot length (mm) per plant ± SD**	**Root dried weight (mg) per plant ± SD**	**Shoot dried weight (mg) per plant ± SD**	**Shoot length (mm) per plant ± SD**	**Shoot dried weight (mg) per plant ± SD**
							**3.5%**	**7%**							
Water	/	/	/	/	/	/	/	/	98.13 ± 3.96	176.50 ± 32.51	135.43 ± 17.57	25.95 ± 6.23	12.45 ± 3.79	285.95 ± 32.79	57.50 ± 14.05
control															
DEF06	0.00 ± 0.07	+	10.75 ± 3.18	1.00 ± 0.00	+	+	+	–	93.75 ± 5.17	193.17 ± 30.66	127.78 ± 26.58	23.09 ± 5.90	12.19 ± 2.12	nt	nt
DEF07	1.36 ± 0.29	–	–	6.00 ± 1.75	+	–	+	+	95.00 ± 7.56	173.88 ± 22.31	136.06 ± 12.54	22.86 ± 8.22	11.04 ± 2.23	287.13 ± 27.03	51.44 ± 10.29
DEF08	0.00 ± 0.09	–	–	0.5 ± 0.71	+	+	+	+	91.25 ± 11.26	174.33 ± 27.89	125.60 ± 10.19	24.80 ± 7.05	21.24 ± 12.22	309.40 ± 28.10	44.28 ± 12.70
DEF09	2.50 ± 0.04	–	–	1.58 ± 0.80	–	+	+	+	97.50 ± 4.63	118.13 ± 42.97^∗^	132.63 ± 13.62	25.29 ± 6.29	14.08 ± 4.40	307.10 ± 21.20	62.20 ± 7.91
DEF13	0.00 ± 1.74	–	–	5.58 ± 2.65	–	+	+	+	87.50 ± 11.65	199.00 ± 14.67	131.56 ± 15.50	28.35 ± 6.67	9.27 ± 4.05	nt	nt
DEF14	0.47 ± 0.65	–	–	2.17 ± 1.04	+	+	+	+	93.75 ± 7.44	168.93 ± 22.21	130.00 ± 21.84	23.61 ± 6.36	10.51 ± 2.39	nt	nt
DEF15	0.85 ± 1.86	–	–	1.08 ± 0.95	+	+	+	+	97.50 ± 4.63	169.22 ± 21.17	122.00 ± 27.86	25.85 ± 5.54	10.73 ± 1.85	nt	nt
DEF16	0.71 ± 0.86	–	–	1.25 ± 1.09	+	–	+	+	96.25 ± 5.17	143.00 ± 28.40	125.30 ± 17.42	25.44 ± 5.86	10.76 ± 1.95	nt	nt
DEF17	0.00 ± 0.32	+	3.50 ± 0.70	3.58 ± 0.52	+	+	+	–	90.00 ± 5.34	143.42 ± 52.81	131.79 ± 16.79	25.92 ± 6.55	9.54 ± 1.80^∗^	nt	nt
DEF18	0.00 ± 0.03	–	4 ± 0.70	2.12 ± 0.53	+	+	+	+	97.50 ± 4.63	166.06 ± 39.08	122.72 ± 25.31	31.03 ± 6.44	12.45 ± 1.60	nt	nt
DEF19	0.96 ± 0.48	–	–	6.41 ± 1.38	+	–	+	+	100.00 ± 0.00	161.65 ± 23.05	119.76 ± 15.88	26.91 ± 5.81	9.76 ± 2.07	301.47 ± 23.92	52.39 ± 10.42
DEF20	0.00 ± 0.21	–	–	3.42 ± 2.45	–	–	+	–	100.00 ± 0.00	155.39 ± 18.76	128.28 ± 16.60	22.09 ± 5.48	10.08 ± 2.06	269.00 ± 31.86	46.49 ± 8.62
DEF21	1.03 ± 1.01	+	–	5.67 ± 0.63	+	+	+	+	97.50 ± 4.63	152.17 ± 38.13	123.83 ± 22.56	30.01 ± 5.84	11.75 ± 2.89	nt	nt
DEF31	1.17 ± 0.73	–	–	1.00 ± 0.00	+	–	+	+	98.75 ± 3.53	169.00 ± 21.05	132.79 ± 9.99	23.56 ± 5.31	10.36 ± 1.84	nt	nt
DEF33	7.51 ± 0.00	–	–	3.17 ± 0.58	+	–	–	–	98.75 ± 3.53	151.47 ± 25.31	133.24 ± 14.57	24.86 ± 6.39	10.87 ± 2.23	nt	nt
DEF39	1.60 ± 0.04	–	–	1.00 ± 0.00	+	+	+	+	100.00 ± 0.00	147.12 ± 30.20	112.53 ± 19.69^∗^	19.71 ± 7.00	9.93 ± 1.93	302.57 ± 14.11	68.21 ± 10.96
DEF40	0.00 ± 0.13	–	–	1.12 ± 0.18	+	+	+	+	98.75 ± 3.53	142.74 ± 32.36	110.58 ± 20.13^∗^	22.51 ± 4.75	10.02 ± 2.37	nt	nt
DEF41	0.00 ± 0.03	–	–	1.33 ± 0.58	–	–	+	+	100.00 ± 0.00	126.94 ± 21.77^∗^	114.78 ± 23.84^∗^	30.86 ± 3.58	12.75 ± 1.31	nt	nt
DEF46	1.12 ± 0.10	–	36.50 ± 0.00	2.66 ± 2.02	+	–	+	–	98.75 ± 3.53	139.78 ± 31.31^∗^	105.50 ± 21.61^∗^	28.45 ± 7.13	10.45 ± 1.75	nt	nt
DEF47	0.00 ± 0.13	–	–	1.83 ± 0.63	+	–	+	+	93.75 ± 14.08	105.00 ± 17.13^∗^	108.75 ± 10.88^∗^	25.38 ± 8.31	10.04 ± 2.94	312.13 ± 23.32	53.04 ± 11.69
DEF48	1.70 ± 0.02	–	–	7.33 ± 1.89	+	–	+	+	100.00 ± 0.00	133.90 ± 40.73^∗^	109.60 ± 22.38^∗^	23.13 ± 4.75	11.70 ± 2.60	311.55 ± 24.61	48.84 ± 9.65

*Underlined are the strains that were not originally isolated as endophytes. ^∗^Indicates significant difference (P < 0.05) from the control assessed with Dunn’s test and Bonferroni correction for multiple comparison. nt Not tested. SD Standard Deviation. +, halo width 1 mm; −, not active. The growth at high salt concentration were compared to control plates (+ grown like control plates; – not grown).*

**TABLE 3:** Screening tests for biocontrol activity against *F. graminearum in vitro* and *in planta* (germination blotter and soil substrate assays).

**Treatment**	**Dual culture assay**	**Germination blotter assay**			**Soil substrate assay**	
	**Mean of growth inhibition (%)**	**FRR protection (%) per plant**	**FFR protection (%) per treatment**	**Infected shoot dried weight (mg) per plant**	**FFR protection (%) per treatment**	**Infected shoot dried weight (mg) per plant**
Water control	–	–	–	10.69 ± 3.15	–	75.14 ± 16.47
DEF06	66.66 ± 6.41	12.14 ± 23.38	17.61	17.71 ± 4.49^∗^	nt	nt
DEF07	74.07 ± 3.70^∧^	38.20 ± 21.41^*∧^	24.24^∧^	16.35 ± 4.78^∗^	61.28^∗^	57.30 ± 11.65^∗^
DEF08	60.49 ± 4.27	15.20 ± 15.12	–45.45^∗∗^	10.81 ± 4.70	–1.12	64.47 ± 13.80
DEF09	59.26 ± 3.70^∧^	42.66 ± 15.21^*∧^	80.86^*∧^	13.89 ± 3.63	46.38^∗^	59.07 ± 12.01^∗^
DEF13	32.1 ± 10.69^∧^	7.87 ± 19.35^∧^	20.45^∧^	11.88 ± 4.90	nt	nt
DEF14	49.38 ± 2.14^∧^	6.57 ± 19.96^∧^	41.18^*∧^	11.66 ± 5.84	nt	nt
DEF15	37.25 ± 3.92^∧^	18.59 ± 22.84^∧^	62.41^*∧^	11.29 ± 2.42	nt	nt
DEF16	56.79 ± 4.28^∧^	38.45 ± 27.18^*∧^	43.61^*∧^	9.61 ± 2.23	nt	nt
DEF17	39.50 ± 5.66	−7.22 ± 13.51	28.57^∗^	10.57 ± 2.54	nt	nt
DEF18	61.73 ± 2.14	18.48 ± 24.57	15.97	7.49 ± 3.01	nt	nt
DEF19	76.54 ± 2.14^∧^	46.74 ± 17.43^*∧^	25.93^∧^	11.08 ± 2.51	39.55	63.19 ± 12.93
DEF20	77.78 ± 0^∧^	27.26 ± 24.45^*∧^	41.23^∧^	11.23 ± 2.02	10.64	59.58 ± 12.42
DEF21	45.68 ± 9.32	35.22 ± 25.73^∗^	12.55	11.86 ± 2.08	nt	nt
DEF31	41.98 ± 19	29.14 ± 20.43^∗^	–14.81	10.98 ± 1.64	nt	nt
DEF33	55.56 ± 0	7.67 ± 21.09	17.36	11.29 ± 1.84	nt	nt
DEF39	64.20 ± 2.14^∧^	39.77 ± 15.00^*∧^	43.75^*∧^	13.40 ± 3.13	–24.47	44.55 ± 13.36^∗^
DEF40	43.21 ± 2.14	24.13 ± 23.23	38.82	8.61 ± 1.53	nt	nt
DEF41	60.49 ± 4.27^∧^	35.96 ± 18.90^*∧^	54.17^*∧^	10.19 ± 2.47	nt	nt
DEF46	39.50 ± 8.55	21.07 ± 25.69	45.00	8.90 ± 2.33	nt	nt
DEF47	54.32 ± 4.28^∧^	24.79 ± 23.31^∧^	87.50^*∧^	9.81 ± 2.63	–7.23	54.93 ± 14.24^∗^
DEF48	70.37 ± 3.70^∧^	32.28 ± 27.81^*∧^	55.88^*∧^	10.69 ± 1.77	29.04	61.37 ± 11.00

**Underlined strains were not originally isolated as endophytes. ^∧^Experimental data obtained from Colombo et al. (2019). nt Not tested. FRR Fusarium root rot. FFR Fusarium foot rot. The average of mycelium growth inhibitions recorded in dual culture assay against F. graminearum strain Fg8/1 is reported. In planta results of germination blotter assay or using soil are displayed as FRR protection (^∗^P < 0.05 is considered significant) and FFR protection (Fisher’s test analysis: ^∗^P < 0.01 treatment considered significantly able to maintain the seedling asymptomatic, ^∗∗^P < 0.01 treatment considered significantly able to increase disease severity). The average of infected shoot weight (mg) for each treatment assessed after 10 or 20 days after seed bacterization or transplant is also reported.**

### Evaluation of PGP Effects in Germination Blotter Assay

Under soilless conditions, none of the tested *Streptomyces* strains significantly altered the germination rate compared to the control plants, which had a germination percentage of around 99 (Table 2). A slight but significant reduction of the germination after seed bacterization was observed only for DEF17. Some strains inhibited the shoot and seminal root length 3 days after the seed bacterization (Supplementary File 2). After 10 days of incubation, an overall attenuation of these negative effects was observed (Table 2) except for DEF41, DEF46, DEF47, and DEF48, which still negatively affected both shoot and seminal root elongation (Table 2).

Ten days after seed bacterization, root number was not significantly different from the control with the exception of DEF41 and DEF09, which showed significantly lower number of roots compared to the control (4 versus 5) (Supplementary File 2).

To assess the potential gain/loss in biomass, root and shoot dry weight were also assessed after 10 days of growth (Table 2). Overall, the effect was minimal and a significantly lower weight was obtained only for shoots in plants treated with DEF17, while root dry weight was not significantly affected.

### Evaluation of Biocontrol Activity in Germination Blotter Assay

FRR was assessed 8 days after the antagonist inoculation. The results confirmed the biocontrol activity observed *in vitro* (Table 3 and Supplementary File 3) with a correlation coefficient of r = 0.5. DEF07, DEF09, DEF16, DEF19, DEF20, DEF21, DEF31, DEF39, DEF41, and DEF48 significantly reduced the necrosis development on wheat roots in comparison with the untreated control (*P* < 0.05), showing up to 46% inhibition of necrosis.

The Fisher’s test analysis of the FFR scores grouped in asymptomatic (0) and symptomatic (1-2-3-4) showed the ability of DEF09 and DEF47 to maintain the seedlings healthy in comparison to the untreated control (*P* = 2.57e-08, *P* = 3.24e-05). Interestingly, seedlings treated with DEF08 showed more severe necrosis (45.45%) at the crown in comparison to the untreated control (*P* = 7.00e-04) (Table 3 and Supplementary File 4). All *P-*values of Fisher’s test analyses are reported in Supplementary File 5.

The strains with a capacity to reduce FRR did not reduce FFR symptoms in the same manner. The best performing strain against FRR was DEF19 (46.74%), while the best performing strain against FFR was DEF47 with protection percentages of 87.50%. Only DEF09 was able to control both symptoms of *F. graminearum* infection with high level of efficiency, resulting in approximately 80% inhibition of FFR development and > 40% in FRR development.

None of the non-endophytic strains showed the ability to effectively reduce the disease severity *in planta* with the exception of DEF31, which showed a partial efficacy against FRR only (29.14% reduction of necrosis extension).

In order to analyze if the BCA treatments were able to counteract the biomass loss following the infection, the shoots from infected seedlings were dried and weighed. Only DEF06 and DEF07 increased significantly the shoot weight compared to the *Fusarium*-treated control (Table 3).

### Biocontrol and PGP Activities in Soil Substrate

To further verify whether the biocontrol and the PGP activities were consistent in a more complex environment – soil, and over a longer period of cultivation, 26 days-, FFR, stem shoot length and dried weight were evaluated for strains showing interesting biocontrol activities: DEF07, DEF09, DEF19, DEF20, DEF39, DEF47, and DEF48. DEF08 was used as negative control.

First, the colonization of inner root tissues by selected *Streptomyces* strains was verified. All root pieces (10/10) of wheat seedlings were extensively colonized by the tested *Streptomyces* strains on WA plates. They showed the ability to move in soil and internally colonize the plant, including DEF08 that was not originally isolated as endophyte.

The use of soil and the longer cultivation period until disease symptom evaluation and PGP analysis led to decreased BCA activity of most of the strains (Table 3 and Supplementary File 6), with the exception of DEF07 and DEF09 which were able to significantly reduce FFR (61 and 46% level of protection, respectively) (Supplementary File 7 for Fisher’s test *P-*values). Plant growth promotion of non-infected plants colonized by the *Streptomyces* spp. strains was not significant for the two parameters analyzed (Table 2). Shoot dried weight of *Fusarium*-infected plants was, however, affected by some strains: DEF07, DEF09, DEF39, and DEF47 lowered the dried biomass in comparison with the *Fusarium*-treated control (Table 3).

### Biocontrol Effects Against FHB Severity in Growth Chamber and Field Conditions

**FIGURE 1:**
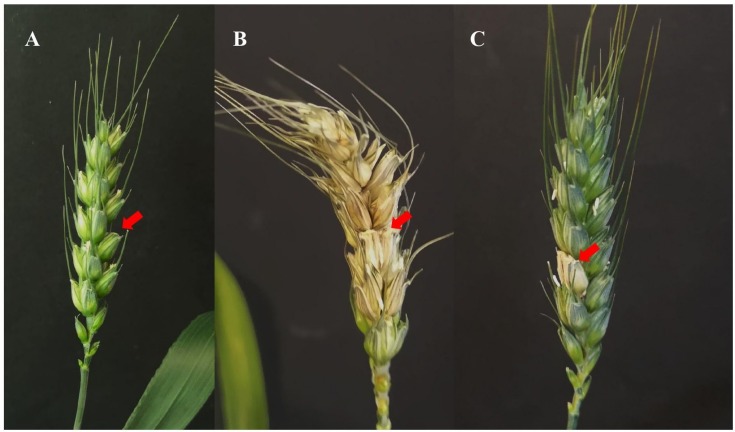
Example of Fusarium head blight symptoms on wheat spikes grown in growth chamber. The red arrow indicates the spikelet of infection. Examples of water inoculated control **(A)**, *Fusarium* inoculated control **(B)** and *Fusarium* + DEF09 treatment **(C)** are shown.

The strain DEF09 showed the best performance against both FRR and FFR diseases, with consistent results in all the assays. Being an endophyte obtained from wheat, its efficacy against FHB disease was first assessed in fully controlled environment (growth chamber). The strain, co-inoculated with the pathogenic strain PH1, stopped the spreading of the disease at the first infected spikelet in all plants (Figure 1). High level of protection (75%) was reached under controlled conditions (Supplementary File 8).

**FIGURE 2:**
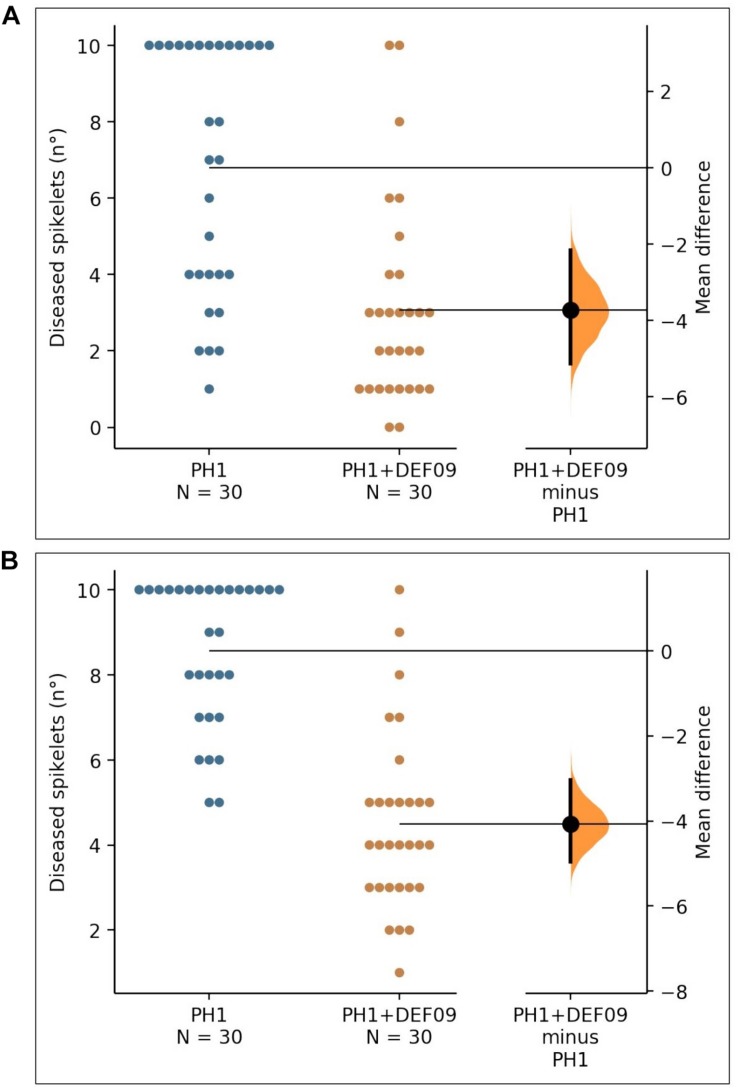
The mean difference between PH1 and PH1 + DEF09 for diseased spikelets (n°) of cultivars “Bandera” **(A)** and “Claudio” **(B)** is shown in the Gardner-Altman estimation plot. The unpaired mean difference of data obtained between PH1 and PH1 + DEF09 is −3.73 (95.0% CI −5.13, −2.17) and −4.07 (95.0% CI −4.97, −3.03) for cv. Bandera and cv. Claudio, respectively. Both groups are plotted on the left axes; the mean difference is plotted on floating axes on the right as a bootstrap sampling distribution. The mean difference is depicted as a dot; the 95% confidence interval is indicated by the vertical error bar.

In order to assess whether the strain could be effective also in field conditions, where different biotic and abiotic interactions occur, a field trial was carried out on bread and durum wheat. The *P-*value of the Mann–Whitney test was 2.47e-05 for Bandera and 1.35e-08 for Claudio. The presence of DEF09 reduced the number of diseased spikelets in comparison to the untreated control (Figure 2), decreasing FHB severity up to 60 and 45% on cv. Bandera and Claudio, respectively (Supplementary File 9).

## Discussion

Comprehensive observation of different parameters, including the physiological characteristics of *Streptomyces* strains and their interaction with the plant (Colombo et al., 2019), is essential for successful selection and characterization of bioactive strains able to adapt to complex environmental conditions and microbiomes (Winter et al., 2019).

In this study, *Streptomyces* strains were extensively characterized for their plant growth associated features, together with detailed examination of their activity on germinating wheat and on wheat infected with *F. graminearum.*

The combination of *in vitro* and *in vivo* laboratory assays led to the identification of an effective strain, DEF09, which also showed promising results in field trials on both durum and bread wheat. The use of FRR and FFR pathosystems for selecting a strain effective against FHB proved successful. This study is in accordance with the observation by Wang L.-Y. et al. (2015), who showed a good correlation between FFR and FHB biocontrol activities for a diverse set of bacterial strains. It also confirms functional analyses of genes from wheat-infecting *Fusarium* species. Different genes were reported to be equally involved in the pathogenic mechanisms of both FHB and FFR (Spanu et al., 2012, 2018; Pasquali et al., 2013). From a physiopathological point of view, *F. graminearum* shows a common infection process during both root- and head infection (Wang Q. et al., 2015; Wang et al., 2018). Our work therefore supports the idea that the use of FRR and FFR pathosystems, being more manageable laboratory models than the FHB pathosystem, is suitable for selection of BCA strains effective against FHB.

In this work it was not possible to include strains previously selected as BCA in other scientific works therefore it is not possible to have a direct comparison of the activity of the strain DEF09 with other *Streptomyces* strains, given that results depend on the complex interactions occurring in the environment (Vurukonda et al., 2018). Nonetheless, based on the reported efficacy of the different microorganisms, the level of protection achieved by the strain DEF09 was comparable to that obtained in field trials using *Bacillus* sp. and *Cryptococcus* sp. (Schisler et al., 2002) and slightly higher than those achieved with other *Streptomyces* strains in field trials on bread wheat (Jung et al., 2013; Palazzini et al., 2017) and durum wheat (Palazzini et al., 2018). It is plausible that the inoculation method may affect the level of protection. Interestingly, Jung et al. (2013) reported significant protection against FHB by the BN1 *Streptomyces* strain only when the strain was sprayed on spikes but not when it was co-inoculated. In our case, the high level of protection, comparable with fungicide treatments (Giraud et al., 2011), was obtained with co-inoculation. Other inoculation methods will need to be tested to better compare the level of protection obtained by DEF09 in different environmental conditions with that of previously studied strains. Novel approaches are also needed to explore the efficacy of the strains in large scale field trials.

The combination of the methods used to assess the bioactivity of the strains examined in our study allowed us to gain insight into their possible mechanisms of activity. For example, the *in vitro* assays carried out on DEF09 suggest that this strain blocks the growth of the fungus with specific antifungal molecules, as shown by the dual culture inhibition assay. Chitinase production has been identified as the main biocontrol mechanism in some studies (Herrera-Estrella and Chet, 1999). DEF09 is a chitinase producer, but the lack of correlation (*r* = 0.22) between the chitinase production in different strains and the growth inhibition of *F. graminearum* indicates that chitin degradation may not be the unique factor responsible for the observed bioactivity of the strains. Likely, the inhibition of fungal growth might be the result of a synergistic effect of different lytic enzymes and metabolites (Zhang and Yuen, 2000; Zhao et al., 2013). DEF09 directly affects wheat plant growth, modifying root development by way of seminal root elongation, as seen in the germination blotter assay after 10 days, and impacting overall plant growth (shoot dried weight) after pathogen infection. Interestingly, morphological changes of roots have been associated with the induction of systemic resistance (Zamioudis et al., 2013). Moreover, DEF09 was among the best IAA producers in the pool. Indeed, IAA is known to play a role in plant morphology as well as in disease modulation, stimulating plant defense (Pieterse et al., 2009) and therefore may contribute to protection against the pathogen. From all these data we may infer that DEF09 possesses multiple mechanisms leading to limitation of FHB on wheat. Metabolic profiling coupled with functional genomics of DEF09 will likely allow for the delineation of the mechanisms of action for the strain (Chen et al., 2018).

A large set of potentially bioactive strains was identified in this study. Strains able to significantly interfere with pathogen development also transiently affected plant growth, suggesting that a complex set of molecules is produced during the tripartite interaction (Mayo-Prieto et al., 2019). Our future goal will be to identify the determinants of these specific interactions occurring among the BCA, the fungus, and the host, as detailed knowledge of their interaction is essential for developing novel plant protection strategies (Berendsen et al., 2012).

## Data Availability Statement

The datasets generated for this study can be found in NCBI GenBank, reported in Table 1. The raw data and additional figures are available in Supplementary Material.

## Author Contributions

MP, EC, MS, and AK contributed to the conception and design of the study. EC, MS, AK, and CP performed the experiments. CP and EC performed the statistical analysis. EC and MP wrote the first draft of the manuscript. AK, MS, PC, and CP wrote sections of the manuscript. All authors contributed to manuscript revision, read and approved the submitted version.

## Conflict of Interest

The authors declare that the research was conducted in the absence of any commercial or financial relationships that could be construed as a potential conflict of interest.
