# Supplementary material for: Selection of an Endophytic Streptomyces sp. Strain DEF09 From Wheat Roots as a Biocontrol Agent Against Fusarium graminearum
Source: Front Microbiol. 2019 Oct 11;10:2356. doi: 10.3389/fmicb.2019.02356 (PMC6798073; doi:10.3389/fmicb.2019.02356)
Supplement: Supplementary file 1 [file Data_Sheet_1.ZIP › Supplementary_files/Supplementary file 5.docx]

| Treatments | *P-value* Fisher test FFR score  0 vs 1-2-3-4 | *P-value* Fisher test FFR score  1-2 vs 3-4 |
| --- | --- | --- |
| DEF06 | 0.1905 | 1 |
| DEF07 | 0.157 | 1 |
| DEF08 | 1 | 0.0007716* |
| DEF09 | 2.57e-08* | 0.5375 |
| DEF13 | 0.0309 | 0.4735 |
| DEF14 | 1 | 3.109E-05* |
| DEF15 | 1 | 1.055E-06* |
| DEF16 | 0.04712 | 0.006847* |
| DEF17 | 1 | 0.001232* |
| DEF18 | 1 | 0.04975 |
| DEF19 | 1 | 0.2273 |
| DEF20 | 0.2117 | 0.2379 |
| DEF21 | 1 | 0.6012 |
| DEF31 | 1 | 1 |
| DEF33 | 1 | 0.2273 |
| DEF39 | 1 | 0.004476* |
| DEF40 | 1 | 0.01704 |
| DEF41 | 1 | 0.0003* |
| DEF46 | 1 | 0.02722 |
| DEF47 | 3.244e-05* | 0.4574 |
| DEF48 | 1 | 0.002728* |
