# Supplementary material for: Selection of an Endophytic Streptomyces sp. Strain DEF09 From Wheat Roots as a Biocontrol Agent Against Fusarium graminearum
Source: Front Microbiol. 2019 Oct 11;10:2356. doi: 10.3389/fmicb.2019.02356 (PMC6798073; doi:10.3389/fmicb.2019.02356)
Supplement: Supplementary file 1 [file Data_Sheet_1.ZIP › Supplementary_files/Supplementary file 7.docx]

| Treatments | *P-value* Fisher test FFR score  0 vs 1-2-3-4 | *P-value* Fisher test FFR score  1-2 vs 3-4 |
| --- | --- | --- |
| DEF07 | 0.287 | 0.01353* |
| DEF08 | 1 | 0.5006 |
| DEF09 | 0.3433 | 0.003892* |
| DEF19 | 0.3068 | 0.2948 |
| DEF20 | 1 | 1 |
| DEF39 | 1 | 0.296 |
| DEF47 | 0.606 | 0.7446 |
| DEF48 | 1 | 0.2767 |
