# Supplementary material for: Selection of an Endophytic Streptomyces sp. Strain DEF09 From Wheat Roots as a Biocontrol Agent Against Fusarium graminearum
Source: Front Microbiol. 2019 Oct 11;10:2356. doi: 10.3389/fmicb.2019.02356 (PMC6798073; doi:10.3389/fmicb.2019.02356)
Supplement: Supplementary file 1 [file Data_Sheet_1.ZIP › Supplementary_files/Supplementary file 8.docx]

| Treatment | Disease severity (%) | Protection level (%) |
| --- | --- | --- |
| PH1 | 63% **±23.1** | - |
| PH1+DEF09 | 15% **±7.5** | 75% |
