# Supplementary material for: Selection of an Endophytic Streptomyces sp. Strain DEF09 From Wheat Roots as a Biocontrol Agent Against Fusarium graminearum
Source: Front Microbiol. 2019 Oct 11;10:2356. doi: 10.3389/fmicb.2019.02356 (PMC6798073; doi:10.3389/fmicb.2019.02356)
Supplement: Supplementary file 1 [file Data_Sheet_1.ZIP › Supplementary_files/Supplementary_caption.docx]

**Supplementary file 1** Example of scale used for FFR symptoms evaluation on wheat seedlings grown in soil: 0 = symptomless; 1 = slightly necrotic; 2 = moderately necrotic; 3 = severely necrotic; 4 = completely necrotic.

**Supplementary file 2** Mean and standard deviation of root and shoot length (mm) for each treatment measured after 3 days post seed bacterization. In addition, mean and standard deviation of root number for each treatment measured after 10 days post seed bacterization are reported. **In bold** values significantly different (*P*<0.05) from those of the control treatment assessed with Dunn’s test and Bonferroni correction for multiple comparison.

**Supplementary file 3** Mean and standard deviation of FRR length (mm) for germination blotter assay measured after 10 days post seed bacterization. **In bold** values significantly different (*P*<0.05) from those of the control treatment assessed with Dunn’s test and Bonferroni correction for multiple comparison.

**Supplementary file 4** FFR scores assessed after 10 days post seed bacterization for each germination blotter assay. In addition disease index, disease severity and level of protection are also reported.

**Supplementary file 5** *P-value* obtained from the two Fisher’s tests performed in R software on FFR data obtained in germination blotter assay. Firstly, scores were pooled in two groups: completely asymptomatic (0 class) versus symptomatic (1-4 classes). In addition, in order to understand if the severity of the symptoms was reduced by the *Streptomyces* treatments a second Fisher’s test was performed comparing the classes 1-2 (mild symptomatic) 3-4 (severely symptomatic). Distribution of scores of the antagonist treatments were compared with the control ones. (*P<0.01 treatment considered significantly able to maintain the seedling asymptomatic).

**Supplementary file 6** FFR scores assessed after 20 days from transplant for soil substrate assay. In addition, disease index, disease severity and level of protection are also reported.

**Supplementary file 7** *P-value* obtained from the two Fisher’s tests performed in R software on FFR data obtained in soil substrate assay. Firstly, scores were pooled in two groups: completely asymptomatic (0 class) versus symptomatic (1-4 classes). In addition, in order to understand if the severity of the symptoms was reduced by the *Streptomyces* treatments a second Fisher’s test was performed comparing the classes 1-2 (mild symptomatic) 3-4 (severely symptomatic). Distribution of scores of the antagonist treatments were compared with the control ones. (*P<0.01 treatment considered significantly able to maintain the seedling asymptomatic)

**Supplementary file 8**. Average of disease severity (%) for each treatment and relative level of protection (%) on FHB disease in controlled conditions.

**Supplementary file 9** Raw data of field trial. The number of diseased spikelets and the corresponding scores using a visual scale (Stack and McMullen, 1998) for each wheat cultivar are reported. In addition, the right table summarize the disease severity (%) for each treatment and the relative level of protection (%).
